# Significance of micro-*EGFR* T790M mutations on EGFR-tyrosine kinase inhibitor efficacy in non-small cell lung cancer

**DOI:** 10.1038/s41598-023-45337-3

**Published:** 2023-11-13

**Authors:** Takeshi Masuda, Satoru Miura, Yuki Sato, Motoko Tachihara, Akihiro Bessho, Atsushi Nakamura, Taichi Miyawaki, Kohei Yoshimine, Masahide Mori, Hideaki Shiraishi, Kosuke Hamai, Koji Haratani, Sumiko Maeda, Eriko Tabata, Chiyoe Kitagawa, Junko Tanizaki, Takumi Imai, Shohei Nogami, Nobuyuki Yamamoto, Kazuhiko Nakagawa, Noboru Hattori

**Affiliations:** 1 Department of Respiratory Medicine, Hiroshima University Hospital, Hiroshima, 734-8551 Japan; 2 Department of Internal Medicine, Niigata Cancer Center Hospital, 2-15-3 Kawagishi-cho, Niigata, 951-8566 Japan; 3 Department of Respiratory Medicine, Kobe City Medical Center General Hospital, Kobe, 650-0047 Japan; 4 Division of Respiratory Medicine, Department of Internal Medicine, Kobe University Graduate School of Medicine, Kobe, 650-0017 Japan; 5 Department of Respiratory Medicine, Japanese Red Cross Okayama Hospital, Okayama, 700-8607 Japan; 6 Department of Pulmonary Medicine, Sendai Kousei Hospital, Sendai, 980-0873 Japan; 7 Division of Thoracic Oncology, Shizuoka Cancer Center, Shunto-gun, 411-8777 Japan; 8 Department of Respiratory Medicine, Iizuka Hospital, Iizuka, 820-8505 Japan; 9 Department of Thoracic Oncology, National Hospital Organization, Osaka Toneyama Medical Center, Toyonaka, 560-8552 Japan; 10 Department of Respiratory Medicine, Mitsui Memorial Hospital, Tokyo, 101-8643 Japan; 11 Department of Respiratory Medicine, Hiroshima Prefectural Hospital, Hiroshima, 734-8530 Japan; 12 Department of Medical Oncology, Kindai University Faculty of Medicine, Osakasayama, 589-8511 Japan; 13 Department of General Thoracic Surgery, Dokkyo Medical University, Shimotsuga-gun, 321-0293 Japan; 14 Department of Respiratory Medicine, Ikeda City Hospital, Ikeda, 563-8510 Japan; 15 Department of Respiratory Medicine and Medical Oncology, National Hospital Organization Nagoya Medical Center, Nagoya, 460-0001 Japan; 16 Department of Medical Oncology, Kishiwada City Hospital, Kishiwada, 596-8501 Japan; 17 Department of Medical Statistics, Osaka Metropolitan University Graduate School of Medicine, Osaka, 558-8585 Japan; 18 Department of Genome Analysis, LSI Medience Corporation, Tokyo, 174-8555 Japan; 19 Department of Internal Medicine III, Wakayama Medical University, Wakayama, 641-8509 Japan

**Keywords:** Medical research, Oncology

## Abstract

Small amounts of epidermal growth factor receptor (*EGFR*) T790M mutation (micro-T790M), which is detected using droplet digital PCR (ddPCR) but not conventional PCR, in formalin-fixed and paraffin-embedded (FFPE) samples have been investigated as a predictive factor for the efficacy of EGFR-tyrosine kinase inhibitors (TKIs). However, the predictive value of micro-T790M remains controversial, possibly owing to the failure to examine artificial T790M in FFPE specimens. Therefore, we examined the predictive value of micro-T790M in first-generation (1G), second-generation (2G), and third-generation (3G) EGFR-TKI efficacy using a new method to exclude FFPE-derived artificial mutations in our retrospective cohort. The primary objective was time to treatment failure (TTF) of 1G, 2G, and 3G EGFR-TKIs according to micro-T790M status. In total, 315 patients with *EGFR*-positive non-small cell lung cancer treated with 1G, 2G, and 3G EGFR-TKIs were included in this study. The proportion of patients positive for micro-T790M in the 1G, 2G, and 3G EGFR-TKI groups was 48.2%, 47.1%, and 47.6%, respectively. In the micro-T790M-positive group, the TTF was significantly longer in the 2G and 3G EGFR-TKI groups than in the 1G TKI group. No differences in the micro-T790M-negative group were observed. Micro-T790M status detected using ddPCR, eliminating false positives, may be a valuable predictor of EGFR-TKI efficacy.

## Introduction

Epidermal growth factor receptor (*EGFR*) gene mutations are the most common driver mutations in patients with lung adenocarcinoma^1^. A third-generation (3G) EGFR tyrosine kinase inhibitor (TKI), osimertinib, is the preferred first-line treatment, although 1st and 2nd generations (1G, and 2G, respectively) EGFR-TKIs have also been recommended^2,3^. The strategy for selecting appropriate first-line treatments for patients with EGFR-mutated non-small cell lung cancer (NSCLC) using concomitant genetic alterations or mutation status remains the major challenge in clinical practice.

*EGFR* mutation detection methods have been developed along with the development of therapeutic agents. Polymerase chain reaction (PCR) methods are conventional standard qualitative methods with a low detection limit (sensitivity) of 1–10%. Conventional PCR methods, including the Cobas *EGFR* Mutation Test v2, Therascreen EGFR assay, and PNA LNA PCR-Clamp, were validated for commercial use and used as diagnostic tools in clinical practice^4–7^. In addition, droplet digital PCR (ddPCR) was developed for the quantitative evaluation of gene mutations with a lower detection limit (< 0.1%) than conventional PCR (1–10%)^8^. Today, several targeted therapeutic drugs for driver mutations are clinically available in addition to EGFR-TKI. Therefore, next-generation sequencing (NGS) is increasingly used to detect driver mutations.

The most common mechanism underlying the acquired resistance in patients treated with 1G and 2G EGFR-TKIs is *EGFR* exon20 T790M mutation^9–11^. In vitro, compared with that of 3G EGFR-TKIs, the 50% inhibitory concentration for T790M-positive cells of 1G and 2G EGFR-TKIs is high and moderate, respectively^12^. In addition, de novo* EGFR* T790M mutations are detected in 0.5–5.8% of EGFR-TKI naïve patients using conventional PCR^13,14^. These previous studies showed that 1G EGFR-TKI is ineffective, whereas 3G EGFR-TKI is effective for patients with de novo T790M mutation detected using conventional PCR^15^.

The significance of very small amounts of *EGFR* T790M mutation with a variant allele frequency (VAF) of less than 1.0% (micro-T790M mutation), which is detected using droplet digital PCR (ddPCR) but not conventional PCR, on EGFR-TKI efficacy has been investigated^16,17^. Similar to the data using conventional PCR, a previous report has shown that a small amount of T790M (micro-T790M mutation) detected using ddPCR is a negative predictive factor for 1G and 2G EGFR-TKIs^17^. Another study has reported better efficacy for 1G EGFR-TKI in patients with micro-T790M mutation than without^16^. Considering these reports, the clinical significance of pretreatment micro-*EGFR* T790M subclones on 1G and 2G EGFR-TKI efficacy has not been clarified.

We hypothesize that the failure to assess the presence of an artificial T790M mutation caused by hydrolytic deamination over time in formalin-fixed and paraffin-embedded (FFPE) specimens may be the cause of these conflicting results^18^. We newly defined micro-T790M mutation by excluding artificial T790M mutations due to hydrolytic deamination^19^. The aim of this retrospective study was to investigate the significance of newly defined micro-T790M mutation in 1G, 2G, and 3G EGFR-TKI efficacy using the large *EGFR* mutation-positive NSCLC cohort.

## Results

### Patients characteristics

**Figure 1:**
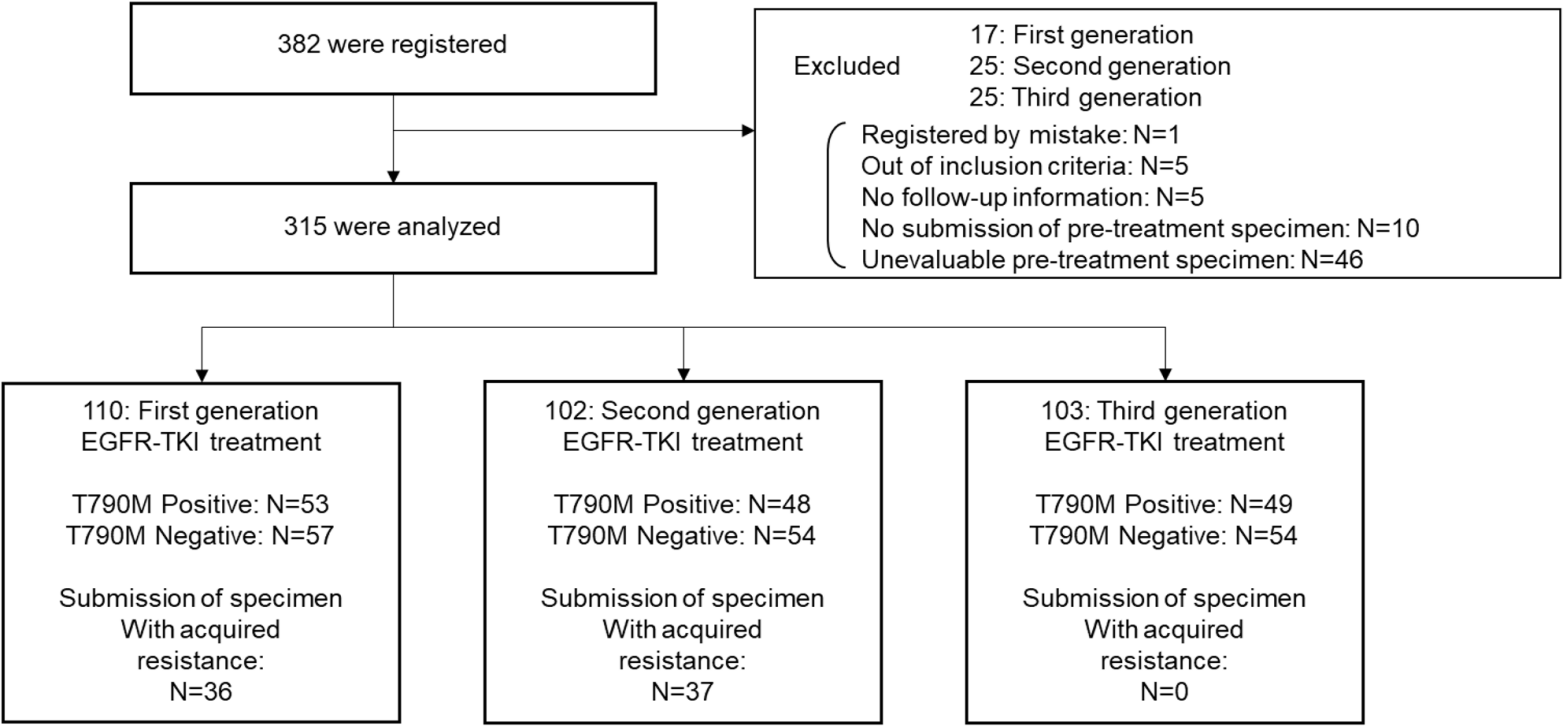
Flowchart of participants. EGFR-TKI, epidermal growth factor receptor-kinase inhibitors.

A total of 382 patients were recruited. A flowchart of the patient selection process is shown in Fig. 1. Ultimately, 315 patients were included in the analysis. A total of 110, 102, and 103 patients received 1G, 2G, and 3G EGFR-TKIs, respectively. Of these patients, 48.2%, 47.1%, and 47.6% were classified as micro-T790M-positive and had received 1G, 2G, and 3G EGFR-TKIs, respectively. The clinical characteristics of the micro-*EGFR* T790M-positive and -negative patients are listed in Table 1. The clinical characteristics of micro-*EGFR* T790M-positive and -negative patients with the Del19 or L858 mutations are shown in Tables S1 and S2.

**Table 1:** Clinical characteristics of micro-T790M-positive and -negative patients.

	First generation			Second generation			Third generation		
	T790M-positive N = 53	T790M-negative N = 57	P value	T790M-positive N = 48	T790M-negative N = 54	P value	T790M-positive N = 49	T790M-negative N = 54	P value
Age (years), Mean ± SD	71.34 ± 10.05	73.60 ± 9.93	0.239	66.50 ± 10.30	66.89 ± 8.91	0.840	72.00 ± 8.45	70.59 ± 9.67	0.433
PS									
0	20 (37.7%)	24 (42.1%)	0.908	23 (47.9%)	29 (53.7%)	0.585	26 (53.1%)	24 (44.4%)	0.393
1	24 (45.3%)	26 (45.6%)		20 (41.7%)	21 (38.9%)		21 (42.9%)	23 (42.6%)	
2	7 (13.2%)	6 (10.5%)		3 (6.2%)	4 (7.4%)		2 (4.1%)	4 (7.4%)	
3	0 (0.0%)	1 (1.8%)		0 (0.0%)	0 (0.0%)		0 (0.0%)	3 (5.6%)	
4	0 (0.0%)	0 (0.0%)		2 (4.2%)	0 (0.0%)		0 (0.0%)	0 (0.0%)	
Missing	2 (3.8%)	0 (0.0%)		0 (0.0%)	0 (0.0%)		0 (0.0%)	0 (0.0%)	
Sex									
Male	22 (41.5%)	18 (31.6%)	0.324	25 (52.1%)	19 (35.2%)	0.110	22 (44.9%)	25 (46.3%)	1.000
Female	31 (58.5%)	39 (68.4%)		23 (47.9%)	35 (64.8%)		27 (55.1%)	29 (53.7%)	
Smoking									
Never	25 (47.2%)	38 (66.7%)	0.050	21 (43.8%)	32 (59.3%)	0.275	35 (71.4%)	29 (53.7%)	0.114
Former	24 (45.3%)	13 (22.8%)		19 (39.6%)	17 (31.5%)		9 (18.4%)	20 (37.0%)	
Current	4 (7.5%)	6 (10.5%)		8 (16.7%)	5 (9.3%)		5 (10.2%)	5 (9.3%)	
Histological type									
Adenocarcinoma	53 (100.0%)	57 (100.0%)	1.000	48 (100.0%)	53 (98.1%)	1.000	48 (98.0%)	53 (98.1%)	1.000
Other	0 (0.0%)	0 (0.0%)		0 (0.0%)	1 (1.9%)		1 (2.0%)	1 (1.9%)	
Stage									
III	4 (7.5%)	5 (8.8%)	1.000	4 (8.3%)	4 (7.4%)	0.808	4 (8.2%)	5 (9.3%)	0.488
IV	35 (66.0%)	38 (66.7%)		34 (70.8%)	36 (66.7%)		28 (57.1%)	36 (66.7%)	
Relapse	14 (26.4%)	14 (24.6%)		10 (20.8%)	14 (25.9%)		17 (34.7%)	13 (24.1%)	
Brain metastasis									
Yes	22 (41.5%)	12 (21.1%)	0.024	11 (22.9%)	12 (22.2%)	1.000	13 (26.5%)	18 (33.3%)	0.522
No	31 (58.5%)	45 (78.9%)		37 (77.1%)	42 (77.8%)		36 (73.5%)	36 (66.7%)	
EGFR-TKI treatment									
Gefitinib	32 (60.4%)	44 (77.2%)	0.066	–	–		–	–	
Erlotinib	21 (39.6%)	13 (22.8%)		–	–		–	–	
Treatment line									
First-line	53 (100.0%)	54 (94.7%)	0.244	46 (95.8%)	51 (94.4%)	1.000	48 (98.0%)	54 (100.0%)	0.476
Second-line	0 (0.0%)	3 (5.3%)		2 (4.2%)	3 (5.6%)		1 (2.0%)	0 (0.0%)	
EGFR exon 19 deletion									
Positive	23 (43.4%)	26 (45.6%)	0.850	30 (62.5%)	40 (74.1%)	0.285	28 (57.1%)	24 (44.4%)	0.238
Negative	30 (56.6%)	31 (54.4%)		18 (37.5%)	14 (25.9%)		21 (42.9%)	30 (55.6%)	
EGFR exon 21 L858R									
Positive	30 (56.6%)	31 (54.4%)	0.850	18 (37.5%)	14 (25.9%)	0.285	21 (42.9%)	30 (55.6%)	0.238
Negative	23 (43.4%)	26 (45.6%)		30 (62.5%)	40 (74.1%)		28 (57.1%)	24 (44.4%)	

Data are presented as mean ± standard deviation or number (percentage).

*PS* performance status, *EGFR-TKI* epidermal growth factor receptor-tyrosine kinase inhibitor.

### ddPCR results

The T790M ratios in the 1G, 2G, and 3G EGFR-TKI groups were 0.269 [0.115–0.870], 0.279 [0.134–0.473], and 0.418 [0.203–0.677], respectively (data are presented as median (%) [interquartile range]) (Supplementary Fig. S1). Specimens obtained after acquired resistance to 1G and 2G EGFR-TKI were available in 36 and 37 cases, respectively (Fig. 1). The micro-*EGFR* T790M ratio in the T790M-positive group detected using the Cobas method was significantly higher than that in the T790M-negative group (Supplementary Fig. S2).

### Comparison of time-to-treatment failure (TTF) in patients treated with 1G, 2G, and 3G EGFR-TKI in micro-*EGFR* T790M-positive and -negative groups

**Figure 2:**
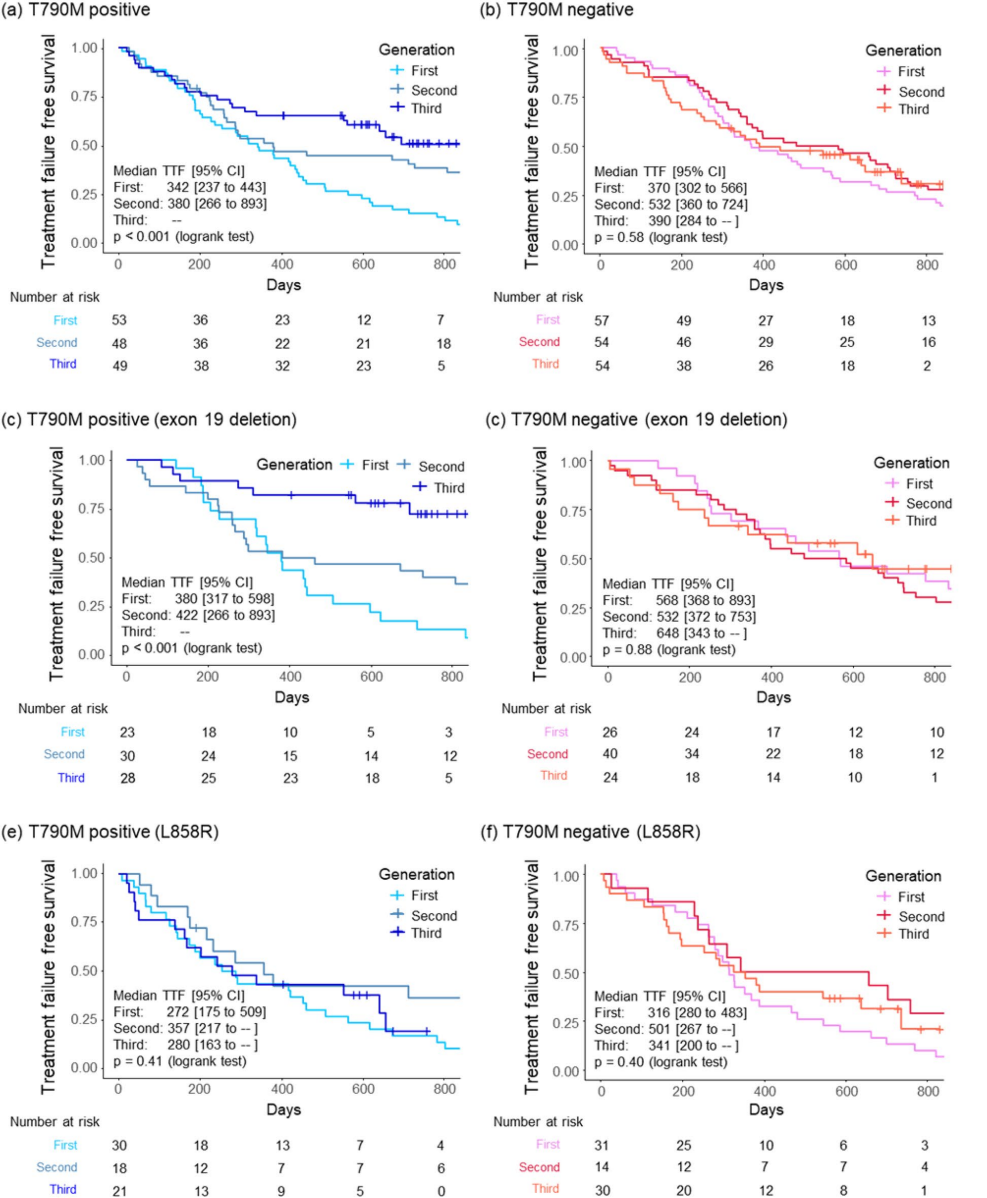
Kaplan–Meier analysis of the time-to-treatment failure between 1G, 2G, and 3G EGFR-TKIs. Patients in the micro-*EGFR* T790M-positive group (**a**) and micro-*EGFR* T790M-negative group (**b**). Patients with EGFR exon 19 deletion in the (**c**) micro-EGFR T790M-positive and (**d**) -negative groups, and patients with EGFR L858R mutation in the (**e**) micro-EGFR T790M-positive and (**f**) -negative groups. *TTF* time-to-treatment failure, *CI* confidence interval, *1G* first-generation, *2G* second-generation, *3G* third generation, *EGFR-TKIs* epidermal growth factor receptor-kinase inhibitors.

The TTFs increased in an ascending order for the patients treated with 1G, 2G, and 3G EGFR-TKIs, respectively, and statistically significant differences were observed (Supplementary Fig. S3a). In addition, the patients treated with 3G EGFR-TK had significantly longer TTFs when compared to the 1G and 2G EGFR-TKI group (Supplementary Fig. S3b). In the micro-*EGFR* T790M-positive group, the TTF in patients treated with 2G and 3G EGFR-TKI was significantly longer than that in patients treated with 1G EGFR-TKI (Fig. 2a). However, these differences were not observed in the micro-*EGFR* T790M-negative subgroup (Fig. 2b). In the sensitivity analysis with T790M ratio cut-offs of 0.001 and 0.003, the proportions of micro-*EGFR* T790M-positive cases for 1G, 2G, and 3G were 38.1%, 38.2%, and 40.8%, respectively, when the T790M ratio cut-off was 0.001 (Supplementary Fig. S4a,b), whereas they were 21.8%, 22.5%, and 31.1%, respectively, when the cut-off was 0.003 (Supplementary Fig. S4c,d); similar results to those observed at the cut-off value of 0 were obtained. In the subgroup analysis based on the *EGFR* mutation status, TTF in patients treated with 3G EGFR-TKI was significantly longer than that of patients treated with 1G and 2G EGFR-TKI in the Del19 and micro-*EGFR* T790M-positive subgroup (Fig. 2c,d). However, these differences were not observed in the L858R mutation subgroup (Fig. 2e,f).

### Comparison of TTF in micro-*EGFR* T790M-positive and -negative patients treated with 1G, 2G, and 3G EGFR-TKIs

**Figure 3:**
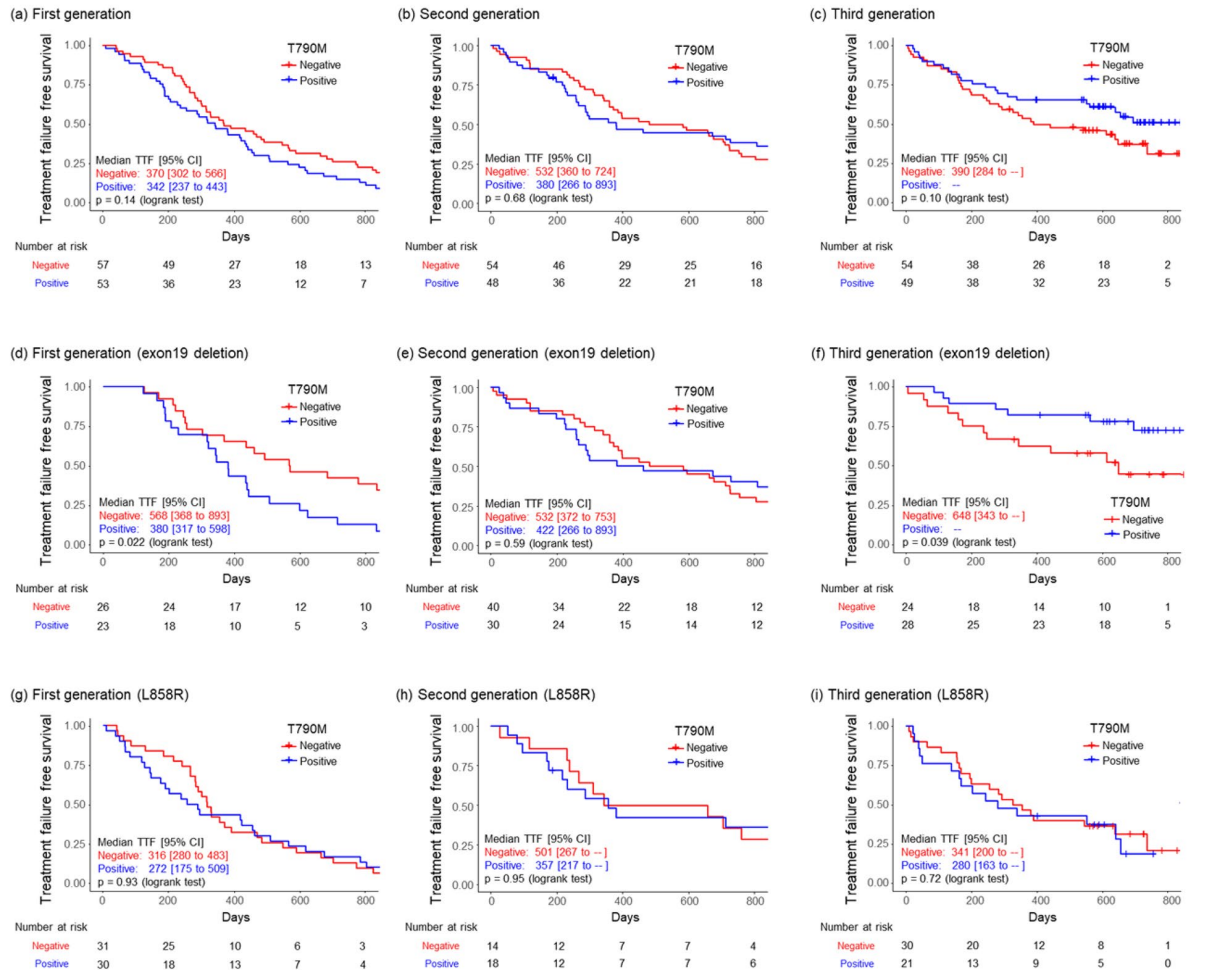
Kaplan–Meier analysis of time-to-treatment failure in micro-*EGFR* T790M-positive and -negative patients. Patients treated with (**a**) 1G, (**b**) 2G, and (**c**) 3G EGFR-TKI. Patients with EGFR exon 19 deletion treated with (**d**) 1G, (**e**) 2G, and (**f**) 3G EGFR-TKI, and patients with EGFR L858R mutation treated with (**g**) 1G, (**h**) 2G, and (**i**) 3G EGFR-TKI. *TTF* time-to-treatment failure, *CI* confidence interval, *1G* first-generation, *2G* second-generation, *3G* third generation, *EGFR* epidermal growth factor receptor.

The TTF in patients treated with 1G EGFR-TKI in the micro-*EGFR* T790M-positive group was shorter than that in the negative group. In contrast, the TTF in patients treated with 3G EGFR-TKI in the micro-*EGFR* T790M-positive group was longer than that in the negative group (Fig. 3a–c). However, these results were not significantly different. In the Del19 subgroup (Fig. 3d–f), the TTF in patients treated with 1G EGFR-TKI in the micro-*EGFR* T790M-positive group was significantly shorter than that in the negative group. In the 3G EGFR-TKI-treated group, the TTF in the micro-*EGFR* T790M-positive group was significantly longer than that in the negative group. Conversely, in the L858R mutation subgroup, these differences were not observed (Fig. 3g–i).

### Multivariable Cox regression analysis of TTF in patients who underwent EGFR-TKI treatment

Multivariable Cox regression analyses showed that performance status, clinical stage, brain metastasis, and *EGFR* mutation status were independent predictors of antitumor effects of EGFR-TKIs. (Table 2). In the micro-*EGFR* T790M-positive group, the 2G (Hazard ratio (HR), [95% confidence interval (CI)]: 0.625, [0.400–0.975], p = 0.038) or 3G (HR, [95% CI)]: 0.495, [0.296–0.829], p = 0.007) EGFR-TKI treatment was an independent predictive factor based on the 1G EGFR-TKI. The following results in the Del19 subgroup are listed in Table S3. In the 3G EGFR-TKI administration group, micro-*EGFR* T790M positivity was an independent predictor (HR, [95% CI)]: 0.388, [0.125–0.990], p = 0.048). Furthermore, in the micro-*EGFR* T790M-positive group, 3G EGFR-TKI was an independent factor based on the 1G (HR, [95% CI)]: 0.231 [0.097–0.549], p = 0.001) and 2G (HR, [95% CI)]: 0.337 [0.142–0.800], p = 0.014) EGFR-TKIs. In contrast, in the L858R subgroup (Table S3), the results observed in the Del 19 subgroup are not shown.

**Table 2:** Multivariable Cox regression analysis of time to treatment failure (full analysis set).

		Hazard ratio	95% CI	P value
PS 2–4 (ref. 0–1)		1.651	[1.102–2.474]	0.015
Male (ref. female)		0.900	[0.656–1.236]	0.517
Current or past smoker (ref. non-smoker)		1.209	[0.883–1.657]	0.236
TNM III or IV (ref. others)		1.879	[1.363–2.589]	< 0.001
Brain metastasis		1.380	[1.027–1.853]	0.032
EGFR exon 19 deletion: positive (ref. EGFR exon 21 L858R: positive)		0.681	[0.523–0.886]	0.004
T790M Positive vs. Negative (ref.)	First generation	1.254	[0.843–1.865]	0.263
T790M Positive vs. Negative (ref.)	Second generation	0.832	[0.534–1.297]	0.417
T790M Positive vs. Negative (ref.)	Third generation	0.760	[0.440–1.314]	0.326
Second generation vs. First generation (ref.)	T790M-negative	0.942	[0.627–1.414]	0.772
Third generation vs. First generation (ref.)	T790M-negative	0.817	[0.524–1.275]	0.374
Third generation vs. Second generation (ref.)	T790M-negative	0.868	[0.547–1.379]	0.549
Second generation vs. First generation (ref.)	T790M-positive	0.625	[0.400–0.975]	0.038
Third generation vs. First generation (ref.)	T790M-positive	0.495	[0.296–0.829]	0.007
Third generation vs. Second generation (ref.)	T790M-positive	0.793	[0.460–1.367]	0.403

*CI* confidence interval, *PS* performance status, *TNM* tumor node metastasis classification, *EGFR* epidermal growth factor receptor.

### Pretreatment micro-*EGFR* T790M mutation ratio in the positive and negative cases calculated via the Cobas method using samples with acquired resistance to EGFR-TKIs

The median pretreatment micro-*EGFR* T790M ratio in the T790M-positive group detected using the Cobas method, represented as median (%) [interquartile range], was 0.061 [0.016–0.39], and that in T790M-negative cases was 0.016 [0.016–0.022] (Supplementary Fig. S5). There were no significant differences between the two groups.

### Cox regression analysis of TTF to evaluate the effects of artificial T790M mutations

We performed Cox proportional hazards analyses to validate the effects of artificial T790M mutations on the efficacy of EGFR-TKIs. In the analysis considering artificial T790M mutations and allele frequency of the WT EGFR gene, the relative hazard of TTF in 1G or 3G EGFR-TKI tended to increase or decrease with the increase in micro-T790M mutation clones (Supplementary Fig. S6a–c). However, this tendency was not observed in the analysis without considering the artificial T790M mutations and allele frequency of the WT EGFR gene (Supplementary Fig. S6d–f) or considering only the allele frequency of the WT EGFR gene (Supplementary Fig. S6g–i).

## Discussion

To the best of our knowledge, this is the first study to show the significance of micro-*EGFR* T790M subclones in 1G, 2G, and 3G EGFR-TKI efficacy, assessed using ddPCR after excluding FFPE-derived artificial mutations. In the micro-*EGFR* T790M-positive group, the efficacy of 2G and 3G EGFR-TKI was significantly higher than that of 1G EGFR-TKI, and the efficacy of 3G EGFR-TKI tended to be superior to that of 2G EGFR-TKI. These results were not observed in the micro-*EGFR* T790M-negative groups. These findings suggest 3G EGFR-TKI as the preferred first-line treatment for micro-*EGFR* T790M-positive NSCLC. Conversely, 1G and 2G EGFR-TKIs may be appropriate first-line treatments for patients without micro-*EGFR* T790M mutation. Administration of sequential treatment from 1G or 2G to 3G EGFR-TKI is recommended after acquired resistance due to T790M mutation. In particular, several observational studies have shown that sequential treatment of initial afatinib followed by osimertinib may provide long-term TTF and overall survival in successful cases, despite problems in managing afatinib toxicity^20^. Further study is needed to determine in which patient groups this treatment strategy is preferable.

The significance of micro-*EGFR* T790M mutations on EGFR-TKIs has been previously investigated using ddPCR^16,17^; however, the predictive value of micro-T790M mutation remains controversial. A previous study showed that the efficacy of 1G EGFR-TKI was higher in patients with micro-T790M than without it^16^, and this is contrary to the preclinical findings showing the efficacy of 1G EGFR-TKI was low on T790M-positive cells^12^. In contrast, our study showed the significance of micro-*EGFR* T790M subclones in EGFR-TKI treatment, which reflect the efficacy of EGFR-TKI against T790M-positive cancer cells as previously demonstrated in a preclinical study^12^. The evaluation of the positivity of micro-*EGFR* T790M mutation after excluding FFPE-derived artificial mutations in our study may be the reason for the discrepancy in results between our study and the previous study^16^. We validated that the significance of micro-*EGFR* T790M mutation on 1G, 2G, and 3G EGFR-TKIs efficacy is correctly examined by excluding FFPE-derived artificial T790M mutations (Supplementary Fig. S4). This result suggests that excluding the FFPE-derived artificial mutations is essential to examine the significance of micro-*EGFR* T790M mutations on the efficacy of EGFR-TKIs.

Using our study’s micro-EGFR T790M-positive criteria, 40–50% of cases were micro-*EGFR* T790M-positive. The positive criteria for micro-*EGFR* T790M have not been established; hence we performed a sensitivity analysis using a different cut-off value to set the proportion of micro-*EGFR* T790M-positive cases as 20–30% and examined the significance of micro-*EGFR* T790M on the efficacy. The effect of micro-*EGFR* T790M on the efficacy of EGFR-TKIs was similar. Several previous studies showed that the proportion of micro-*EGFR* T790M subclones varied significantly in each report (8.0–79.9%)^16,17,21–23^. The false-positive T790M allele derived from the FFPE-artificial mutation and the different cut-off points for micro-*EGFR* T790M positivity might have affected these data. We propose that the cut-off value and positive criteria for micro-*EGFR* T790M positivity in our study, but the appropriate criteria for microT790M positivity to predict the EGFR-TKI efficacy needs further prospective study.

Our study showed the significance of micro-*EGFR* T790M in Del19 but not in the L858R subgroup. TTF in patients treated with 3G EGFR-TKI was significantly longer than that in patients treated with 1G and 2G EGFR-TKI in the Del19 but not L858R and micro-EGFR T790M mutation-positive groups. These results may be strongly influenced by the presence of co-mutation (tumor suppressor mutations), which is detected in EGFR-mutated NSCLC. The presence of co-mutation has been reported to attenuate the effect of EGFR-TKI in L858R but not Del19-positive cases^24^. Furthermore, a previous study using NGS has shown that more compound mutations that can affect the therapeutic effect of EGFR-TKI is detected in L858R than in Del19^25^. We believe that the presence of co-mutation and compound mutation affecting the efficacy of EGFR-TKI is the reasons why we could not detect the significance of mT790M in the L858R subgroup in our study. We are conducting a phase III trial to evaluate the superiority of erlotinib plus ramucirumab over osimertinib in EGFR L858R-positive patients, and we plan to investigate the significance of micro-T790M in this trial^26^.

In this study, we report the significance of treatment selection based on the micro T790M clone, which is detected by ddPCR and not the PCR methods used in clinical practice. As the number of driver gene mutations increases, NGS-based multi-detection systems have become mainstream in practical clinical practice^2,27^. The sensitivity of NGS for T790M detection is lower than that of PCR^8,28^, and it is assumed that the micro T790M clones shown in the present study are difficult to detect by NGS. Whether micro T790M detected by NGS can help in the selection of treatment for EGFR mutated lung cancer needs to be further investigated.

Presently, it is important to collect tumor specimens of sufficient quality and quantity and to evaluate adequate driver mutations^29^. There are a few caveats to detect microT790M by the ddPCR method used in this study. First, tumor tissues properly fixed and stored for as short a time as possible were needed to minimize DNA degradation^30^. Second, large biopsy tumor samples with a tumor content percentage of 10% or greater or surgically resected samples would be needed to perform ddPCR analysis^30^. Further studies are needed to determine the appropriate quality and quantity of tumor tissue required for micro-T790M analysis to adapt this method to real-world practice.

EGFR T790M mutation clones are generated through two theories: clonal selection and evolution^31–33^. To determine which theory is associated with our results, we compared the micro-*EGFR* T790M ratio in T790M-positive and -negative cases detected using the Cobas method and found that the micro-*EGFR* T790M ratio was not significantly different between the two groups. We believe that this result supports the “clonal evolution theory.” However, the small size and heterogeneity of the tumors^34^ might have contributed to this result. The results in the present study indicate that it is difficult to estimate the acquired T790M mutation after 1G or 2G EGFR-TKI treatment using the information about the presence of pretreatment micro-*EGFR* T790M.

This study has some limitations. First, this was a retrospective study. Second, concurrent genetic alterations, including TP53 and BIM polymorphism, have been reported as factors affecting the efficacy of EGFR-TKIs^35^. Our study did not examine the effects of these factors on EGFR-TKI efficacy.

In conclusion, this study shows that micro-*EGFR* T790M mutations influence EGFR-TKI efficacy in patients with NSCLC. Detection of micro-*EGFR* T790M mutations using ddPCR and eliminating FFPE-derived false positive mutation will be helpful in selecting optimal EGFR-TKI regimens for patients with *EGFR-*mutated NSCLC.

## Methods

### Study population and design

We enrolled patients with unresectable NSCLC who harbored *EGFR* Del19 or L858R without the T790M mutation, which was verified using conventional PCR methods. Patients from 31 Japanese institutions who received 1G or 2G EGFR-TKIs until June 2018, or 3G EGFR-TKIs until June 2019, as first- or second-line treatment were consecutively included. The exclusion criteria were patients with *EGFR* Del19 or L858R with other *EGFR* mutations and those whose EGFR-TKI treatment was changed from one generation to another owing to the lack of tumor response. This retrospective study was approved by the institutional review board of Hiroshima University (No. E939) and the ethics committee of each participating institution. The opt-out method of patient consent for participation was applied in this study and written informed consent was obtained from all patients as per the guidance of the ethics committee of several institutions. The registration number of the study is UMIN000040474. All methods were performed following the relevant guidelines and regulations.

### Data collection and outcomes

We collected the following data from medical records: age, sex, smoking history, clinical stage, Eastern Cooperative Oncology Group performance status, histologic type, presence or absence of brain metastasis, line of treatment, the status of EGFR mutation, and type of EGFR-TKIs. The primary outcome of this study was TTF, defined as the time from EGFR-TKI treatment initiation to death, disease progression, or treatment discontinuation.

### ddPCR

ddPCR was performed using pre-EGFR-TKI treatment FFPE tumor tissue specimens obtained after April 2015 (LSI Medience Corporation, Tokyo, Japan), as described in the Supplementary Doc (Droplet digital polymerase chain reaction (ddPCR)). To validate the quality of our experimental system, ddPCR was performed using normal genomic 50 ng DNA (Horizon, Tokyo, Japan) and DNA with T790M mutations (Horizon) in each concentration. A correlation was observed between the concentration of the sample and the measured mutation (Supplementary Fig. S7).

### Inclusion criteria of samples for analysis

Our ddPCR using the normal genomic DNA (Horizon) and DNA derived from the peripheral blood of healthy volunteers showed one T790M-positive (FAM+ and HEX−) drop in one DNA sample (Table S4). Therefore, to exclude samples with this artificial positive drop, we included samples with two or more FAM+ and HEX− drops for the analysis set. Subsequently, cases with a (FAM+ and HEX−) drop from 0 to 1 were included in the analysis set when more than 2000 wild-type drops were detected. The flow chart for analysis is shown in Supplementary Fig. S8. Cases involving the amplification of the *EGFR* mutations were excluded. The criteria for amplification are described in Supplementary Doc (The criteria of EGFR gene amplification) and Supplementary Fig. S9.

### Criteria for positive micro-*EGFR* T790M mutation

The cytosine to thymidine (C-to-T) base-pair change within the CpG site at position 790 results in a transition from threonine to methionine (T790M)^9,10^. This cytosine within the CpG site is converted to 5-methylcytosine by methylation^36^. The 5-methylcytosine is converted to thymidine through hydrolytic deamination in the FFPE sample over time. Finally, a false-positive C-to-T change is detected as T790M using PCR. Therefore, to estimate this false-positive T790M mutation, we used the following new method^19^. We considered that cytosine and guanine (CG) were changed to thymine and guanine (TG) in T790; CG in F795, located within the CpG site, was also changed to TG. The primers and probes for mutant F795F and wild-type (WT) F795F (Supplementary Doc (Primer and probes for mutant F795F or WT F795F)) were generated by the LSI Medience Corporation (Tokyo, Japan). ddPCR using the genomic DNA obtained from healthy volunteers showed minimal FAM+ and HEX− drop (Table S5). In addition, the previous experiment showed that deamination by heat treatment induced similar allele frequencies of T790M and F795F in the genomic DNA^19^. Therefore, analysis of the F795F mutation was reasonable for estimating artificial false-positive T790M mutations. In our study, the criteria for micro-T790M positivity were as follows: (VAF of T790M) − (VAF of F795F) > 0. Furthermore, to exclude allele frequency of the WT *EGFR* gene in normal epithelial cells, we determined the final T790M ratio as follows: (VAF of T790M − VAF of F795F)/VAF of active mutation (Del19 or L858R). The lowest value was set to 0.

### Statistical analysis

Patients without follow-up information or submission of the pretreatment specimen were excluded from the analysis. Baseline demographic and clinical characteristic data were expressed as numbers (percentages) for categorical variables and mean ± standard deviation for continuous variables. TTF was depicted using the Kaplan–Meier curves. Differences in TTF among micro-T790M-positive/-negative and treatment generations were assessed using log-rank tests. To evaluate the treatment effects based on the micro-T790M result for each treatment generation with adjustment of known prognostic factors, multivariable Cox proportional hazards analyses were performed. The following variables were included in the models of hazards for treatment failure: performance status, sex, smoking status, cancer type, brain metastasis, indicators of an EGFR exon 19 deletion-positive or EGFR exon 21 L858R-positive result, micro-T790M result, and treatment generation. Analyses were also performed for predefined subgroups of patients positive for EGFR exon 19 deletion and patients positive for EGFR exon 21 L858R. The relationship between the results obtained using the Cobas method and the T790M mutation ratio via ddPCR using samples obtained after acquired resistance to 1G or 2G EGFR-TKIs were examined using the Wilcoxon test. Similarly, the relationship between the results obtained by the Cobas method using samples obtained after acquired resistance to 1G or 2G EGFR-TKIs and the T790M mutation ratio via ddPCR using pretreatment samples were examined using the Wilcoxon test.

The sensitivity analysis for differences in TTF among treatment generations was conducted using T790M ratio cut-offs of 0.001 and 0.003. We performed an exploratory analysis to assess the relationship between micro-T790M values and hazards for treatment failure by Cox proportional hazards analyses. Micro-T790M values were included in the models through the restricted cubic spline function with three knots. The estimated relative hazards for treatment failure were plotted against the micro-T790M values for each treatment generation. Statistical significance for all tests was defined as a two-tailed p-value of ≤ 0.05. Data analyses were performed using R, version 4.1.0 (R Foundation for Statistical Computing, Vienna, Austria, https://www.r-project.org/).

### Supplementary Information

Supplementary Information.

## Data Availability

The data that support the findings of this study are available from the corresponding author upon reasonable request.
