# Supplementary material for: Significance of micro-EGFR T790M mutations on EGFR-tyrosine kinase inhibitor efficacy in non-small cell lung cancer
Source: Sci Rep. 2023 Nov 13;13:19729. doi: 10.1038/s41598-023-45337-3 (PMC10643699; doi:10.1038/s41598-023-45337-3)
Supplement: Supplementary file 1 — Supplementary Information. [file 41598_2023_45337_MOESM1_ESM.docx]

**Significance of micro-*EGFR* T790M mutations on EGFR-tyrosine kinase inhibitor efficacy in non-small cell lung cancer**

Takeshi Masuda^a^, Satoru Miura^b*^, Yuki Sato^c^, Motoko Tachihara^d^, Akihiro Bessho^e^, Atsushi Nakamura^f^, Taichi Miyawaki^g^, Kohei Yoshimine^h^, Masahide Mori^i^, Hideaki Shiraishi^j^, Kosuke Hamai^k^, Koji Haratani^l^, Sumiko Maeda^m^, Eriko Tabata^n^, Chiyoe Kitagawa^o^, Junko Tanizaki^p^, Takumi Imai^q^, Shouhei Nogami^r^, Nobuyuki Yamamoto^s^, Kazuhiko Nakagawa^l^, Noboru Hattori^a^

^a^Department of Respiratory Medicine, Hiroshima University Hospital, Hiroshima 734-8551, Japan

^b^Department of Internal Medicine, Niigata Cancer Center Hospital, Niigata 951-8566, Japan

^c^Department of Respiratory Medicine, Kobe City Medical Center General Hospital, Kobe 650-0047, Japan

^d^Division of Respiratory Medicine, Department of Internal Medicine, Kobe University Graduate School of Medicine, Kobe 650-0017, Japan

^e^Department of Respiratory Medicine, Japanese Red Cross Okayama Hospital, Okayama 700-8607, Japan

^f^Department of Pulmonary Medicine, Sendai Kousei Hospital, Sendai 980-0873, Japan

^g^Division of Thoracic Oncology, Shizuoka Cancer Center, Shunto-gun, 411-8777, Japan

^h^Department of Respiratory Medicine, Iizuka Hospital, Iizuka 820-8505, Japan

^i^Department of Thoracic Oncology, National Hospital Organization, Osaka Toneyama Medical Center, Toyonaka 560-8552, Japan

^j^Department of Respiratory Medicine, Mitsui Memorial Hospital, Tokyo 101-8643, Japan

^k^Department of Respiratory Medicine, Hiroshima Prefectural Hospital, Hiroshima 734-8530, Japan

^l^Department of Medical Oncology, Kindai University Faculty of Medicine, Higashiosaka 577-8502, Japan

^m^Department of General Thoracic Surgery, Dokkyo Medical University, Shimotsuga-gun 321-0293, Japan

^n^Department of Respiratory Medicine, Ikeda City Hospital, Ikeda 563-8510, Japan

^o^Department of Respiratory Medicine and Medical Oncology, National Hospital Organization Nagoya Medical Center, Nagoya, 460-0001, Japan

^p^Department of Medical Oncology, Kishiwada City Hospital, Kishiwada 596-8501, Japan

^q^Department of Medical Statistics, Osaka Metropolitan University Graduate School of Medicine, Osaka 558-8585, Japan

^r^Genome Research Group, Genome Analysis Department, Medical Solution Segment, LSI Medience Corporation, Tokyo 101-8517, Japan

^s^Department of Internal Medicine III, Wakayama Medical University, Wakayama 641-8509, Japan

*Corresponding author: Satoru Miura

Department of Internal Medicine, Niigata Cancer Center Hospital, 2-15-3 Kawagishi-cho, Niigata 951-8566, Japan

Tel: +81-25-266-5111

Fax: +81-25-266-5112

Email: miusat1118@niigata-cc.jp

**Supplementary Figures**

**Figure S1. T790M ratio.**


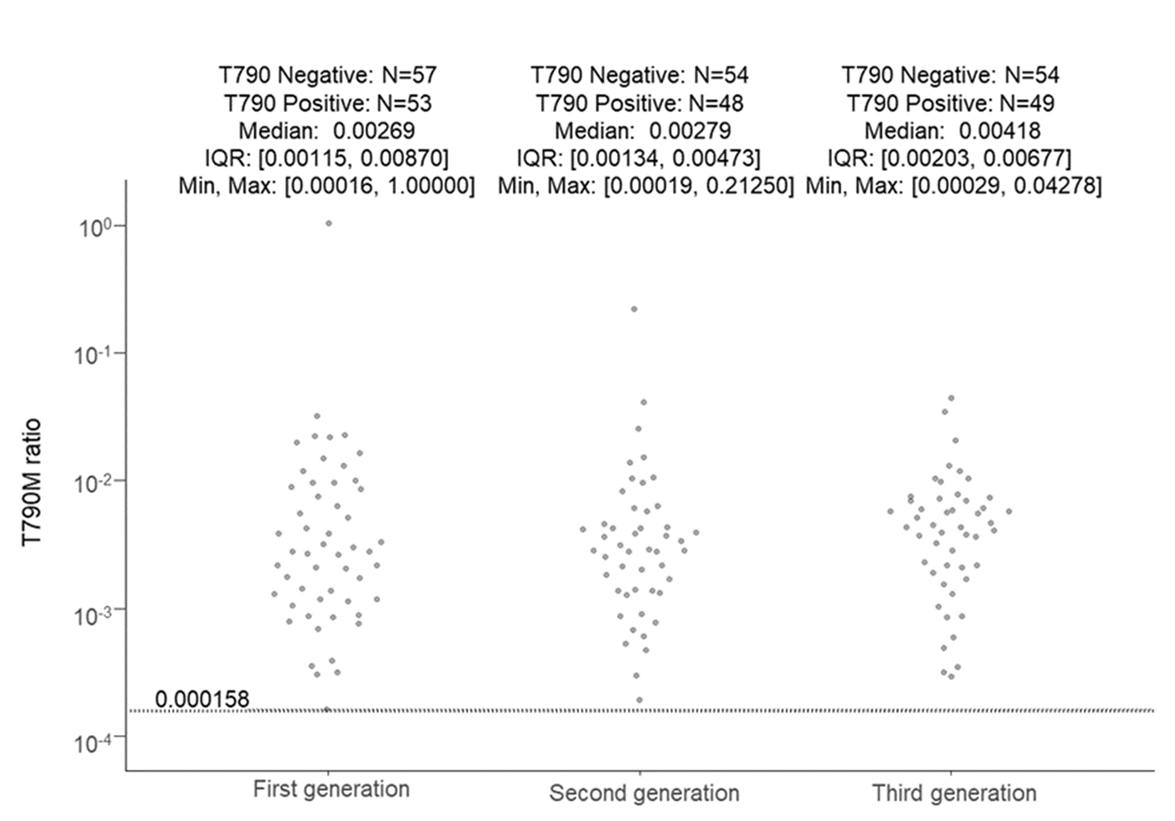


IQR, interquartile range; min, minimum; max, maximum

**Figure S2. Comparison of the T790M mutation ratio via droplet digital PCR in T790M-positive or -negative cases using the Cobas method and samples obtained after acquired resistance to epidermal growth factor receptor-kinase inhibitors.**


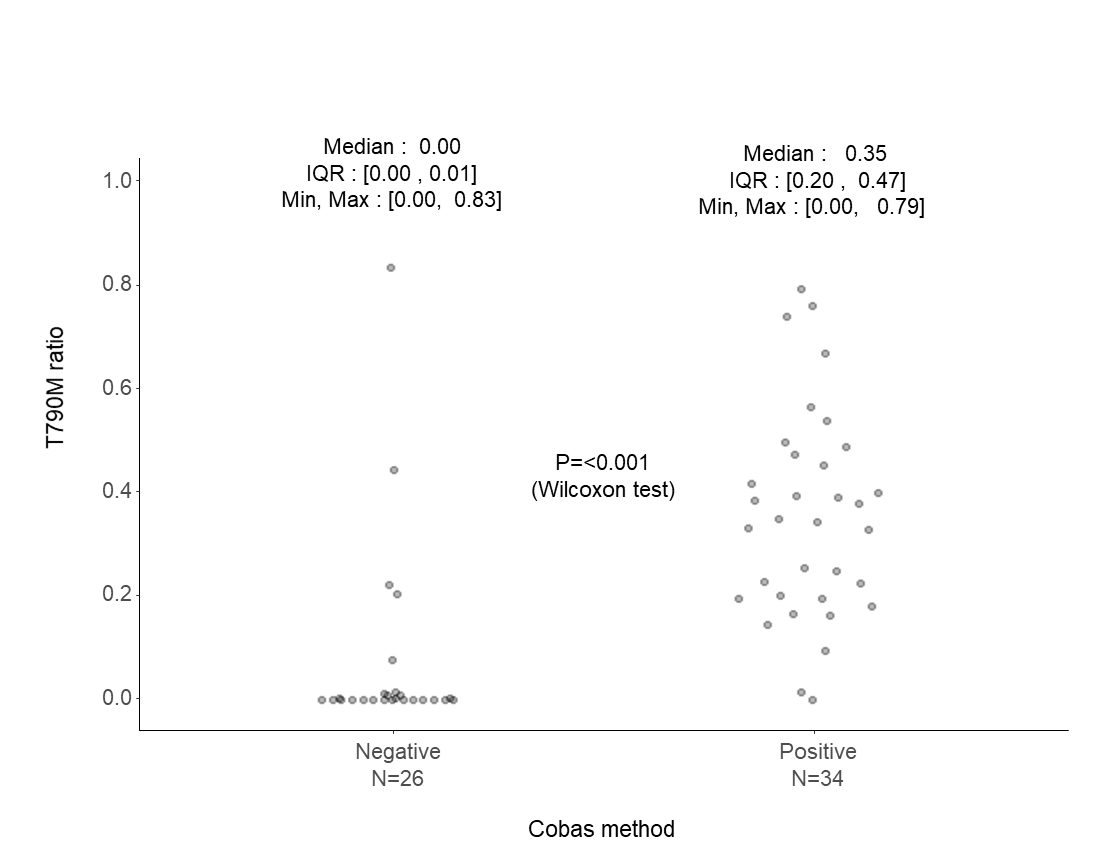


PCR, polymerase chain reaction; IQR, interquartile range; min, minimum; max, maximum

**Figure S3. Comparison of time-to-treatment failure in 1G, 2G, and 3G EGFR-TKI patients**

**
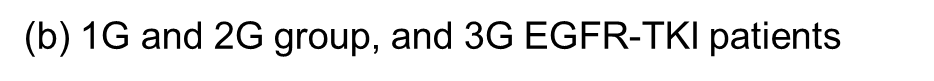

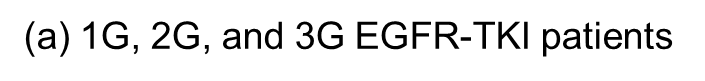
**


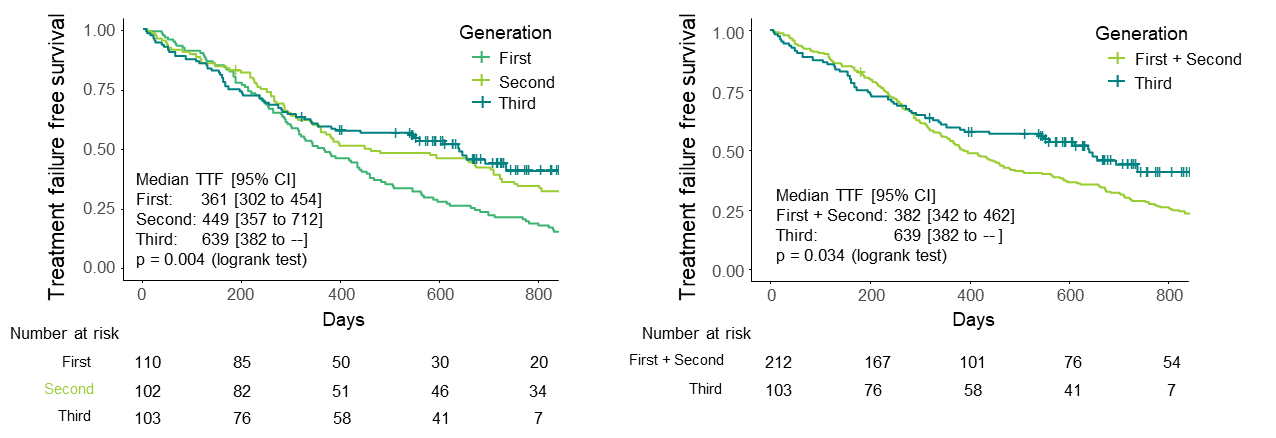


Analysis for differences in TTF (a) between 1G, 2G, and 3G EGFR-TKIs (b) 1G and 2G EGFR-TKIs group and 3G EGFR-TKI.

EGFR, epidermal growth factor receptor; 1G, first-generation; 2G, second-generation; 3G, third generation; EGFR-TKI, epidermal growth factor receptor-kinase inhibitors; TTF, time-to-treatment failure; CI, confidence interval

**Figure S4. Sensitivity analysis for time-to-treatment failure in 1G, 2G, and 3G EGFR-TKI patients in the micro-EGFR T790M-positive and -negative groups in the context of T790M ratio cut-off.**

**
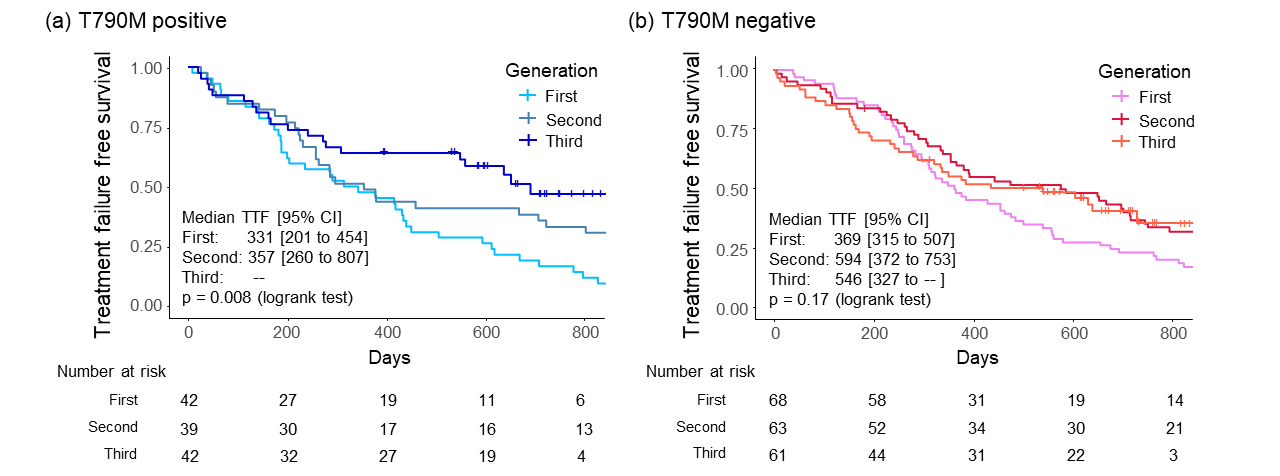
**


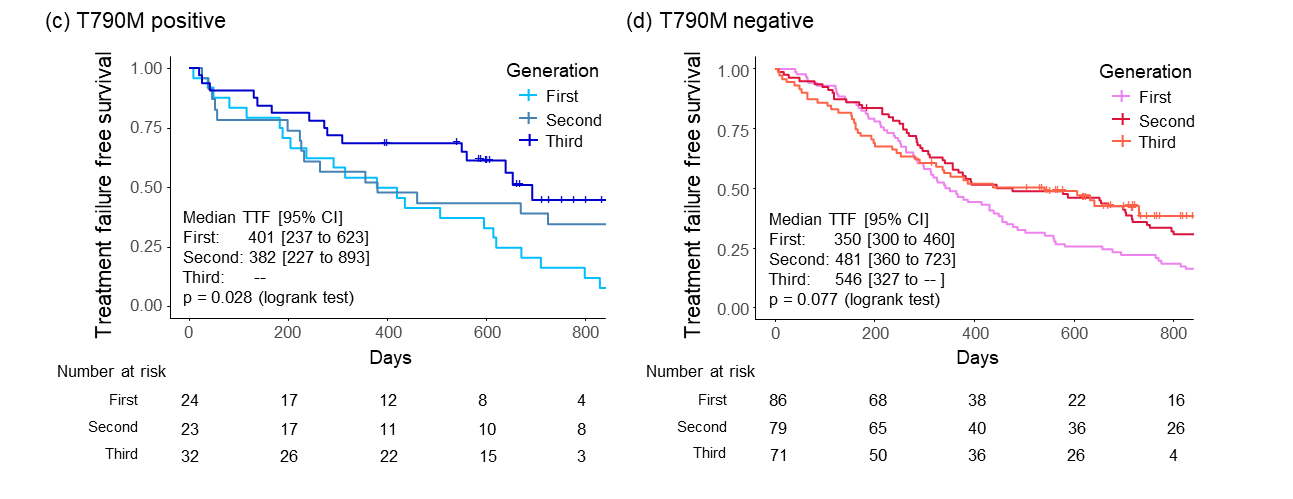


Analysis for differences in TTF among treatment generations (T790M ratio cut-off; 0.001) in the (a) micro-EGFR T790M-positive group and (b) -negative group. The analysis for differences in TTF among treatment generations (T790M ratio cut-off; 0.003) in the (c) micro-EGFR T790M-positive group and (d) -negative group.

EGFR, epidermal growth factor receptor; 1G, first-generation; 2G, second-generation; 3G, third generation; EGFR-TKI, epidermal growth factor receptor-kinase inhibitors; TTF, time-to-treatment failure; CI, confidence interval

**Figure S5. Comparison between the T790M mutation ratio detected via ddPCR in pretreatment samples in T790M-positive and -negative cases using the Cobas method and samples obtained after acquired resistance.**


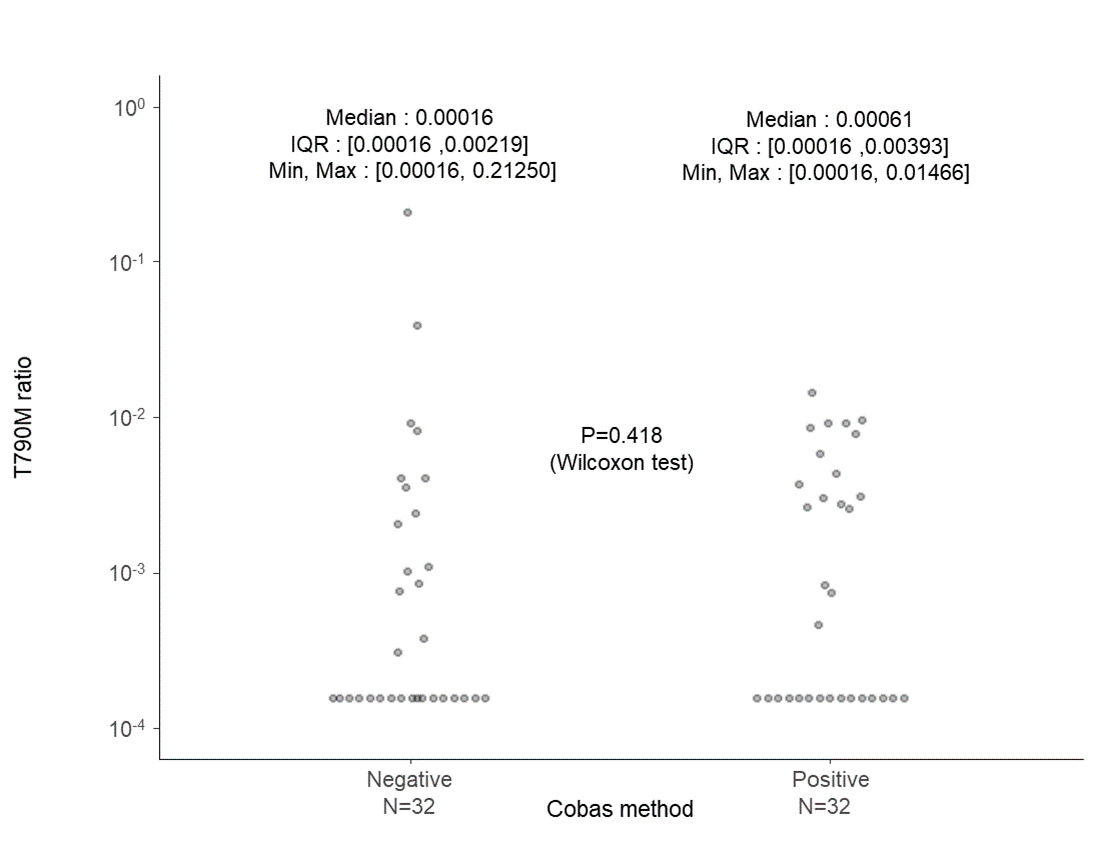


IQR, interquartile range; ddPCR, droplet digital polymerase chain reaction; min, minimum; max, maximum

**Figure S6. Relationship between micro-T790M values and hazards for treatment failure via Cox proportional hazards analyses.**


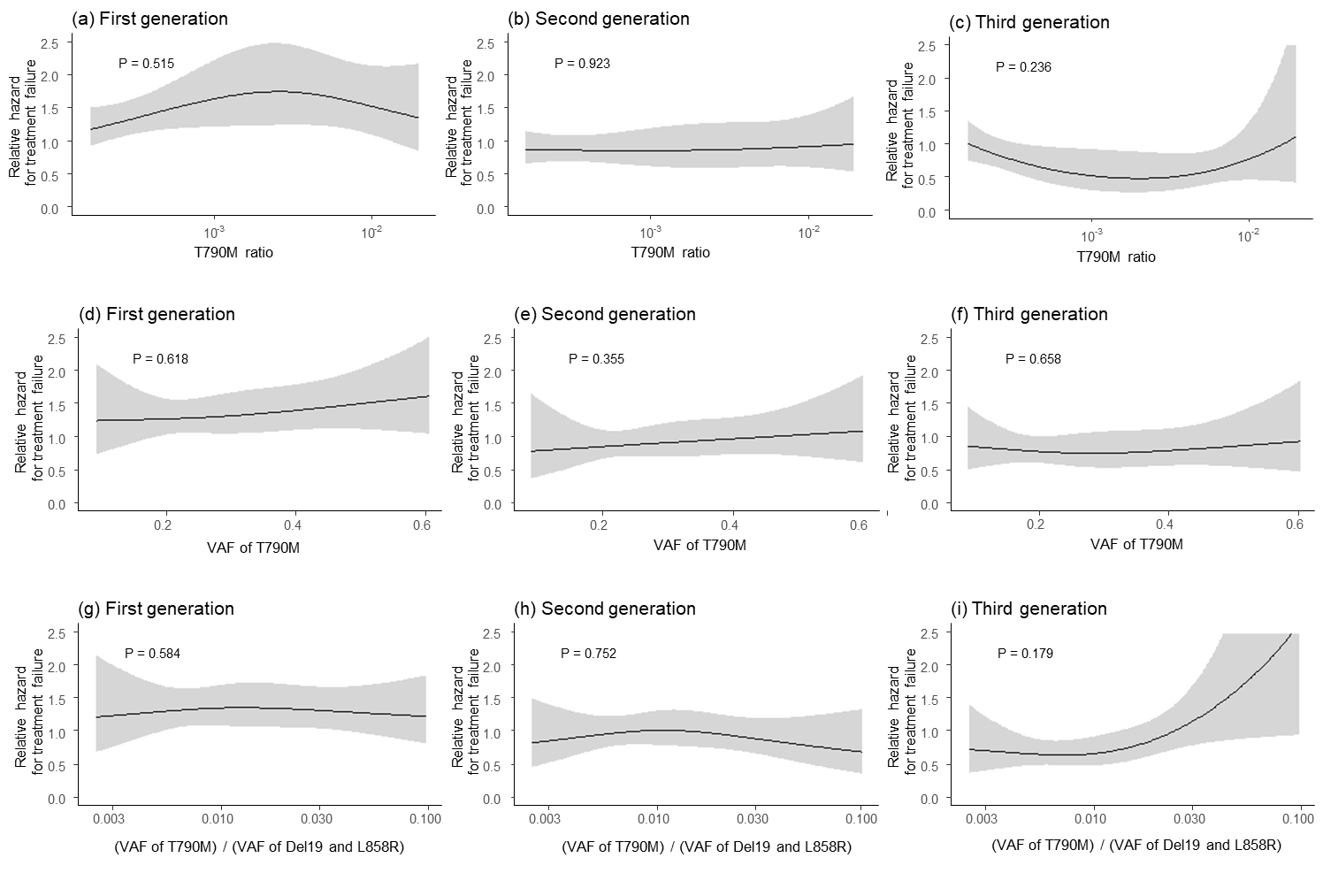


Relationship between (a) T790M ratio, (b) VAF of T790M, (c) (VAF of T790M)/(VAF of Del19 and L858R), and the hazards for treatment failure

VAF, variant allele frequency

**Figure S7. Correlation between the concentration of T790M in the sample and the measured T790M mutation using droplet digital PCR.**

PCR, polymerase chain reaction

**Figure S8. Flowchart of sample selection criteria.**

**
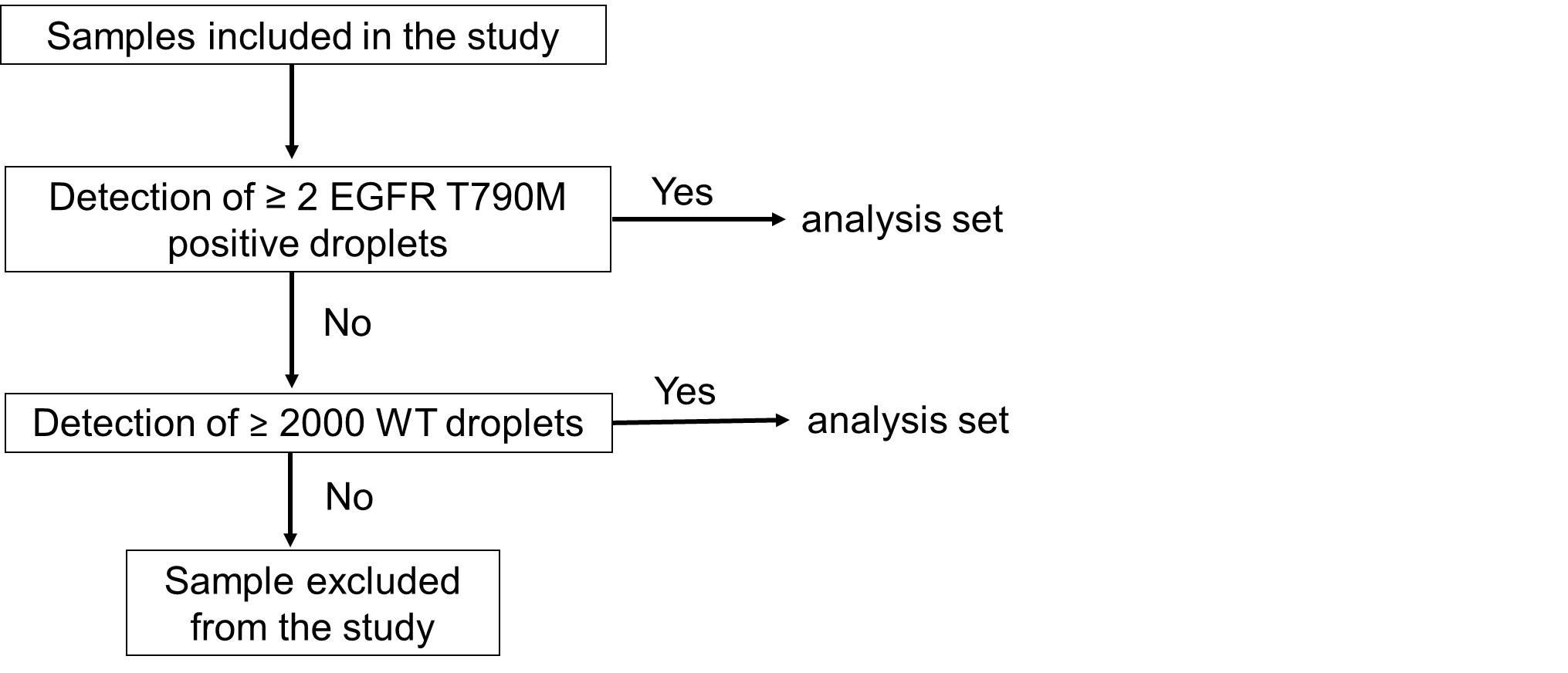
**

EGFR, epidermal growth factor; WT, wild type

**Figure S9. Copy number of samples using droplet digital PCR.**

PCR, polymerase chain reaction

**Supplementary Tables**

**Table S1. Clinical characteristics of micro-T790M-positive and -negative patients with the Del19 mutation.**

|  |  | **First generation** | | | **Second generation** | | | **Third generation** | | | |
| --- | --- | --- | --- | --- | --- | --- | --- | --- | --- | --- | --- |
|  |  | T790M- positive  N=23 | T790M- negative N=26 | P value | T790M- positive  N=30 | T790M- negative N=40 | P value | T790M- positive  N=28 | T790M- negative N=24 | | P value |
| Age (years) |  | 66.96 ± 11.39 | 72.12 ± 11.30 | 0.119 | 64.83 ± 12.07 | 67.70 ± 8.81 | 0.277 | 71.57 ± 8.01 | 70.54 ± 10.55 | | 0.698 |
| PS | 0 | 6 (26.1%) | 14 (53.8%) | 0.016 | 12 (40.0%) | 21 (52.5%) | 0.546 | 17 (60.7%) | 11 (45.8%) | | 0.467 |
|  | 1 | 11 (47.8%) | 11 (42.3%) |  | 15 (50.0%) | 17 (42.5%) |  | 10 (35.7%) | 10 (41.7%) | |  |
|  | 2 | 5 (21.7%) | 0 (0.0%) |  | 2 (6.7%) | 2 (5.0%) |  | 1 (3.6%) | 1 (4.2%) | |  |
|  | 3 | 0 (0.0%) | 1 (3.8%) |  | 0 (0.0%) | 0 (0.0%) |  | 0 (0.0%) | 2 (8.3%) | |  |
|  | 4 | 0 (0.0%) | 0 (0.0%) |  | 1 (3.3%) | 0 (0.0%) |  | 0 (0.0%) | 0 (0.0%) | |  |
|  | Missing | 1 (4.4%) | 0 (0.0%) |  | 0 (0.0%) | 0 (0.0%) |  | 0 (0.0%) | 0 (0.0%) | |  |
| Sex | Male | 9 (39.1%) | 8 (30.8%) | 0.564 | 17 (56.7%) | 16 (40.0%) | 0.227 | 11 (39.3%) | 11 (45.8%) | | 0.779 |
|  | Female | 14 (60.9%) | 18 (69.2%) |  | 13 (43.3%) | 24 (60.0%) |  | 17 (60.7%) | 13 (54.2%) | |  |
| Smoking | Never | 11 (47.8%) | 16 (61.5%) | 0.187 | 12 (40.0%) | 22 (55.0%) | 0.465 | 19 (67.9%) | 13 (54.2%) | | 0.453 |
|  | Former | 12 (52.2%) | 8 (30.8%) |  | 12 (40.0%) | 13 (32.5%) |  | 6 (21.4%) | 9 (37.5%) | |  |
|  | Current | 0 (0.0%) | 2 (7.7%) |  | 6 (20.0%) | 5 (12.5%) |  | 3 (10.7%) | 2 (8.3%) | |  |
| Histological type | Adenocarcinoma | 23 (100.0%) | 26 (100.0%) | 1.000 | 30 (100.0%) | 40 (100.0%) | 1.000 | 28 (100.0%) | 24 (100.0%) | | 1.000 |
|  | Other | 0 (0.0%) | 0 (0.0%) |  | 0 (0.0%) | 0 (0.0%) |  | 0 (0.0%) | 0 (0.0%) | |  |
| Stage | III | 2 (8.7%) | 2 (7.7%) | 0.817 | 2 (6.7%) | 3 (7.5%) | 1.000 | 1 (3.6%) | 3 (12.5%) | | 0.214 |
|  | IV | 16 (69.6%) | 16 (61.5%) |  | 21 (70.0%) | 27 (67.5%) |  | 17 (60.7%) | 17 (70.8%) | |  |
|  | Relapse | 5 (21.7%) | 8 (30.8%) |  | 7 (23.3%) | 10 (25.0%) |  | 10 (35.7%) | 4 (16.7%) | |  |
| Brain metastasis | Yes | 8 (34.8%) | 4 (15.4%) | 0.183 | 5 (16.7%) | 9 (22.5%) | 0.764 | 9 (32.1%) | 7 (29.2%) | | 1.000 |
|  | No | 15 (65.2%) | 22 (84.6%) |  | 25 (83.3%) | 31 (77.5%) |  | 19 (67.9%) | 17 (70.8%) | |  |
| EGFR-TKI treatment | Gefitinib | 13 (56.5%) | 22 (84.6%) | 0.055 | — | — |  | — | — | |  |
|  | Erlotinib | 10 (43.5%) | 4 (15.4%) |  | — | — |  | — | — | |  |
| Treatment line | First line | 23 (100.0%) | 23 (88.5%) | 0.237 | 30 (100.0%) | 39 (97.5%) | 1.000 | 28 (100.0%) | 24 (100.0%) | | 1.000 |
|  | Second line | 0 (0.0%) | 3 (11.5%) |  | 0 (0.0%) | 1 (2.5%) |  | 0 (0.0%) | 0 (0.0%) | |  |
| EGFR exon 19 deletion | Positive | 23 (100.0%) | 26 (100.0%) | 1.000 | 30 (100.0%) | 40 (100.0%) | 1.000 | 28 (100.0%) | 24 (100.0%) | | 1.000 |
|  | Negative | 0 (0.0%) | 0 (0.0%) |  | 0 (0.0%) | 0 (0.0%) |  | 0 (0.0%) | 0 (0.0%) | |  |
| EGFR exon 21 L858R | Positive | 0 (0.0%) | 0 (0.0%) | 1.000 | 0 (0.0%) | 0 (0.0%) | 1.000 | 0 (0.0%) | 0 (0.0%) | | 1.000 |
|  | Negative | 23 (100.0%) | 26 (100.0%) |  | 30 (100.0%) | 40 (100.0%) |  | 28 (100.0%) | 24 (100.0%) | |  |
| Data are presented as mean ± standard deviation or number (percentage).  Abbreviations: EGFR-TKI: epidermal growth factor receptor - tyrosine kinase inhibitor: PS, performance status | | | | | | | | | |  |  |

**Table S2. Clinical characteristics of micro-T790M-positive and -negative patients with the L858R mutation.**

|  |  | **First generation** | | | **Second generation** | | | **Third generation** | | | |  |
| --- | --- | --- | --- | --- | --- | --- | --- | --- | --- | --- | --- | --- |
|  |  | T790M- positive  N=30 | T790M- negative N=31 | P value | T790M- positive  N=18 | T790M- negative N=14 | P value | T790M- positive  N=21 | T790M- negative N=30 | | P value | |
| Age (years) |  | 74.70 ± 7.46 | 74.84 ± 8.62 | 0.947 | 69.28 ± 5.63 | 64.57 ± 9.10 | 0.105 | 72.57 ± 9.18 | 70.63 ± 9.10 | | 0.460 | |
| PS | 0 | 14 (46.7%) | 10 (32.3%) | 0.271 | 11 (61.1%) | 8 (57.1%) | 0.926 | 9 (42.9%) | 13 (43.3%) | | 0.860 | |
|  | 1 | 13 (43.3%) | 15 (48.4%) |  | 5 (27.8%) | 4 (28.6%) |  | 11 (52.4%) | 13 (43.3%) | |  | |
|  | 2 | 2 (6.7%) | 6 (19.4%) |  | 1 (5.6%) | 2 (14.3%) |  | 1 (4.8%) | 3 (10.0%) | |  | |
|  | 3 | 0 (0.0%) | 0 (0.0%) |  | 0 (0.0%) | 0 (0.0%) |  | 0 (0.0%) | 1 (3.3%) | |  | |
|  | 4 | 0 (0.0%) | 0 (0.0%) |  | 1 (5.6%) | 0 (0.0%) |  | 0 (0.0%) | 0 (0.0%) | |  | |
|  | Missing | 1 (3.3%) | 0 (0.0%) |  | 0 (0.0%) | 0 (0.0%) |  | 0 (0.0%) | 0 (0.0%) | |  | |
| Sex | Male | 13 (43.3%) | 10 (32.3%) | 0.434 | 8 (44.4%) | 3 (21.4%) | 0.266 | 11 (52.4%) | 14 (46.7%) | | 0.779 | |
|  | Female | 17 (56.7%) | 21 (67.7%) |  | 10 (55.6%) | 11 (78.6%) |  | 10 (47.6%) | 16 (53.3%) | |  | |
| Smoking | Never | 14 (46.7%) | 22 (71.0%) | 0.112 | 9 (50.0%) | 10 (71.4%) | 0.459 | 16 (76.2%) | 16 (53.3%) | | 0.236 | |
|  | Former | 12 (40.0%) | 5 (16.1%) |  | 7 (38.9%) | 4 (28.6%) |  | 3 (14.3%) | 11 (36.7%) | |  | |
|  | Current | 4 (13.3%) | 4 (12.9%) |  | 2 (11.1%) | 0 (0.0%) |  | 2 (9.5%) | 3 (10.0%) | |  | |
| Histological type | Adenocarcinoma | 30 (100.0%) | 31 (100.0%) | 1.000 | 18 (100.0%) | 13 (92.9%) | 0.438 | 20 (95.2%) | 29 (96.7%) | | 1.000 | |
|  | Other | 0 (0.0%) | 0 (0.0%) |  | 0 (0.0%) | 1 (7.1%) |  | 1 (4.8%) | 1 (3.3%) | |  | |
| Stage | III | 2 (6.7%) | 3 (9.7%) | 0.651 | 2 (11.1%) | 1 (7.1%) | 0.856 | 3 (14.3%) | 2 (6.7%) | | 0.592 | |
|  | IV | 19 (63.3%) | 22 (71.0%) |  | 13 (72.2%) | 9 (64.3%) |  | 11 (52.4%) | 19 (63.3%) | |  | |
|  | Relapse | 9 (30.0%) | 6 (19.4%) |  | 3 (16.7%) | 4 (28.6%) |  | 7 (33.3%) | 9 (30.0%) | |  | |
| Brain metastasis | Yes | 14 (46.7%) | 8 (25.8%) | 0.114 | 6 (33.3%) | 3 (21.4%) | 0.694 | 4 (19.0%) | 11 (36.7%) | | 0.221 | |
|  | No | 16 (53.3%) | 23 (74.2%) |  | 12 (66.7%) | 11 (78.6%) |  | 17 (81.0%) | 19 (63.3%) | |  | |
| EGFR-TKI treatment | Gefitinib | 19 (63.3%) | 22 (71.0%) | 0.592 | — | — |  | — | — | |  | |
|  | Erlotinib | 11 (36.7%) | 9 (29.0%) |  | — | — |  | — | — | |  | |
| Treatment line | First line | 30 (100.0%) | 31 (100.0%) | 1.000 | 16 (88.9%) | 12 (85.7%) | 1.000 | 20 (95.2%) | 30 (100.0%) | | 0.412 | |
|  | Second line | 0 (0.0%) | 0 (0.0%) |  | 2 (11.1%) | 2 (14.3%) |  | 1 (4.8%) | 0 (0.0%) | |  | |
| EGFR exon 19 deletion | Positive | 0 (0.0%) | 0 (0.0%) | 1.000 | 0 (0.0%) | 0 (0.0%) | 1.000 | 0 (0.0%) | 0 (0.0%) | | 1.000 | |
|  | Negative | 30 (100.0%) | 31 (100.0%) |  | 18 (100.0%) | 14 (100.0%) |  | 21 (100.0%) | 30 (100.0%) | |  | |
| EGFR exon 21 L858R | Positive | 30 (100.0%) | 31 (100.0%) | 1.000 | 18 (100.0%) | 14 (100.0%) | 1.000 | 21 (100.0%) | 30 (100.0%) | | 1.000 | |
|  | Negative | 0 (0.0%) | 0 (0.0%) |  | 0 (0.0%) | 0 (0.0%) |  | 0 (0.0%) | 0 (0.0%) | |  | |
| Data are presented as mean ± standard deviation or number (percentage).  Abbreviations: EGFR-TKI: epidermal growth factor receptor - tyrosine kinase inhibitor, PS: performance status | | | | | | | | | |  |  |  |

**Table S3. Multivariable Cox regression analysis of time to treatment failure in Del19- and L858-positive groups.**

|  |  | **Hazard ratio** | **95% CI** | **P value** |
| --- | --- | --- | --- | --- |
| Del19-positive group |  |  |  |  |
| PS 2–4 (ref. 0–1) |  | 2.323 | [1.249 to 4.323] | 0.008 |
| Male (ref. Female) |  | 0.788 | [0.509 to 1.218] | 0.283 |
| Current or past smoker (ref. Non-smoker) |  | 1.531 | [1.001 to 2.342] | 0.049 |
| TNM III or IV (ref. others) |  | 2.235 | [1.396 to 3.580] | 0.001 |
| Brain metastasis |  | 1.456 | [1.081 to 1.961] | 0.013 |
| T790M Positive vs, Negative (ref.) | First generation | 1.242 | [0.654 to 2.358] | 0.508 |
| T790M Positive vs. Negative (ref.) | Second generation | 0.783 | [0.454 to 1.348] | 0.377 |
| T790M Positive vs. Negative (ref.) | Third generation | 0.388 | [0.152 to 0.990] | 0.048 |
| Second generation vs. First generation (ref.) | T790M Negative | 1.084 | [0.633 to 1.857] | 0.768 |
| Third generation vs. First generation (ref.) | T790M Negative | 0.739 | [0.360 to 1.515] | 0.408 |
| Third generation vs. second generation (ref.) | T790M Negative | 0.681 | [0.348 to 1.334] | 0.263 |
| Second generation vs. First generation (ref.) | T790M Positive | 0.684 | [0.362 to 1.289] | 0.240 |
| Third generation vs. First generation (ref.) | T790M Positive | 0.231 | [0.097 to 0.549] | 0.001 |
| Third generation vs. second generation (ref.) | T790M Positive | 0.337 | [0.142 to 0.800] | 0.014 |
| L858R-positive group |  |  |  |  |
| PS 2–4 (ref. 0–1) |  | 1.490 | [0.816 to 2.724] | 0.195 |
| Male (ref. Female) |  | 1.005 | [0.608 to 1.661] | 0.985 |
| Current or past smoker (ref. Non-smoker) |  | 1.038 | [0.622 to 1.730] | 0.887 |
| TNM III or IV (ref. others) |  | 1.716 | [1.079 to 2.729] | 0.023 |
| Brain metastasis |  | 1.219 | [0.776 to 1.918] | 0.390 |
| T790M Positive vs. Negative (ref.) | First generation | 1.148 | [0.658 to 2.003] | 0.627 |
| T790M Positive vs. Negative (ref.) | Second generation | 0.952 | [0.432 to 2.097] | 0.904 |
| T790M Positive vs. Negative (ref.) | Third generation | 1.339 | [0.669 to 2.677] | 0.409 |
| Second generation vs. First generation (ref.) | T790M Negative | 0.695 | [0.350 to 1.379] | 0.298 |
| Third generation vs. First generation (ref.) | T790M Negative | 0.831 | [0.463 to 1.492] | 0.536 |
| Third generation vs. Second generation (ref.) | T790M Negative | 1.196 | [0.566 to 2.530] | 0.639 |
| Second generation vs. First generation (ref.) | T790M Positive | 0.576 | [0.292 to 1.137] | 0.112 |
| Third generation vs. First generation (ref.) | T790M Positive | 0.969 | [0.491 to 1.912] | 0.928 |
| Third generation vs. second generation (ref.) | T790M Positive | 1.682 | [0.778 to 3.635] | 0.186 |

Abbreviations: CI: confidence interval, PS: performance status, TNM: tumor node metastasis classification, EGFR: epidermal growth factor receptor

**TableS 4. Droplet digital PCR for T790M using normal genomic DNA derived from healthy human peripheral blood or commercially available DNA.**

| **Sample** | **Target** | **Copies per  20-uL well** | **Fractional Abundance (%)** | **Positives** | **Negatives** | **Mutant+, Wild+** | **Mutant+, Wild-** | **Mutant-, Wild+** | **Mutant-, Wild-** |
| --- | --- | --- | --- | --- | --- | --- | --- | --- | --- |
|  |  |  |  |  |  | **FAM+HEX+** | **FAM+HEX-** | **FAM-HEX+** | **FAM-HEX-** |
| 1 | Mutant | 6.8 | 0.050 | 3 | 10370 | 3 | 0 | 4373 | 5997 |
|  | Wild | 12900 |  | 4376 | 5997 | 3 | 0 | 4373 | 5997 |
| 2 | Mutant | 5 | 0.035 | 3 | 14221 | 3 | 0 | 6485 | 7736 |
|  | Wild | 14340 |  | 6488 | 7736 | 3 | 0 | 6485 | 7736 |
| 3 | Mutant | 1.4 | 0.012 | 1 | 15694 | 1 | 0 | 6340 | 9354 |
|  | Wild | 12180 |  | 6341 | 9354 | 1 | 0 | 6340 | 9354 |
| 4 | Mutant | 5 | 0.037 | 3 | 14273 | 3 | 0 | 6188 | 8085 |
|  | Wild | 13380 |  | 6191 | 8085 | 3 | 0 | 6188 | 8085 |
| 5 | Mutant | 8.6 | 0.070 | 6 | 16402 | 6 | 0 | 6999 | 9403 |
|  | Wild | 13100 |  | 7005 | 9403 | 6 | 0 | 6999 | 9403 |
| 6 | Mutant | 9 | 0.070 | 5 | 13200 | 5 | 0 | 5789 | 7411 |
|  | Wild | 13600 |  | 5794 | 7411 | 5 | 0 | 5789 | 7411 |
| 7 | Mutant | 7.2 | 0.050 | 4 | 13149 | 4 | 0 | 5945 | 7204 |
|  | Wild | 14160 |  | 5949 | 7204 | 4 | 0 | 5945 | 7204 |
| 8 | Mutant | 8.2 | 0.070 | 5 | 14483 | 5 | 0 | 5775 | 8708 |
|  | Wild | 11980 |  | 5780 | 8708 | 5 | 0 | 5775 | 8708 |
| 9 | Mutant | 3.6 | 0.023 | 2 | 12855 | 1 | 1 | 6323 | 6532 |
|  | Wild | 15920 |  | 6324 | 6533 | 1 | 1 | 6323 | 6532 |
| 10 | Mutant | 1.8 | 0.012 | 1 | 12487 | 1 | 0 | 5956 | 6531 |
|  | Wild | 15260 |  | 5957 | 6531 | 1 | 0 | 5956 | 6531 |
| 11 | Mutant | 1.6 | 0.054 | 1 | 15195 | 1 | 0 | 1807 | 13388 |
|  | Wild | 2980 |  | 1808 | 13388 | 1 | 0 | 1807 | 13388 |
| 12 | Mutant | 0 | 0 | 0 | 14915 | 0 | 0 | 1797 | 13118 |
|  | Wild | 3020 |  | 1797 | 13118 | 0 | 0 | 1797 | 13118 |

Sample 1–8; 50 ng DNA from healthy human peripheral blood

Sample 9, 10; 50 ng commercially available DNA

Sample 11, 12; 10 ng commercially available DNA

PCR: polymerase chain reaction

**Table S5. Droplet digital PCR for F795F using normal genome DNA derived from healthy human peripheral blood.**

| **Sample** | **Target** | **Copies per  20-uL well** | **Fractional Abundance (%)** | **Positives** | **Negatives** | **Mutant+, Wild+** | **Mutant+, Wild-** | **Mutant-, Wild+** | **Mutant-, Wild-** |
| --- | --- | --- | --- | --- | --- | --- | --- | --- | --- |
|  |  |  |  |  |  | **FAM+HEX+** | **FAM+HEX-** | **FAM-HEX+** | **FAM-HEX-** |
| 1 | Mutant | 4.4 | 0.032 | 3 | 16248 | 2 | 1 | 7174 | 9074 |
|  | Wild | 13700 |  | 7176 | 9075 | 2 | 1 | 7174 | 9074 |
| 2 | Mutant | 3 | 0.020 | 2 | 16192 | 2 | 0 | 7505 | 8687 |
|  | Wild | 14660 |  | 7507 | 8687 | 2 | 0 | 7505 | 8687 |
| 3 | Mutant | 6 | 0.045 | 4 | 15692 | 4 | 0 | 6823 | 8869 |
|  | Wild | 13440 |  | 6827 | 8869 | 4 | 0 | 6823 | 8869 |
| 4 | Mutant | 4.2 | 0.035 | 3 | 17206 | 2 | 1 | 6783 | 10423 |
|  | Wild | 11800 |  | 6785 | 10424 | 2 | 1 | 6783 | 10423 |
| 5 | Mutant | 0 | 0 | 0 | 14785 | 0 | 0 | 6699 | 8086 |
|  | Wild | 14200 |  | 6699 | 8086 | 0 | 0 | 6699 | 8086 |
| 6 | Mutant | 3.2 | 0.025 | 2 | 14722 | 2 | 0 | 6148 | 8574 |
|  | Wild | 12720 |  | 6150 | 8574 | 2 | 0 | 6148 | 8574 |

PCR: polymerase chain reaction

**Supplementary Documents**

**Droplet digital polymerase chain reaction (ddPCR)**

Genomic DNA extraction was conducted using the QIAamp DNA Micro Kit (Qiagen, Hilden, Germany) according to the manufacturer's recommendations. Extracted DNA was quantified using a NanoDrop ND-1000 spectrophotometer (Thermo Fisher Scientific, Cleveland, OH, USA). The presence of the *EGFR* mutation was assessed using QX200 AutoDG ddPCR System (Bio-Rad, Hercules, CA, USA). Forward and reverse gene-specific primers and fluorescent hydrolysis probes for either the mutant (Del19, L858R, and T790M-FAM) or wild type (WT) (WT Del19, L858R, and T790-HEX) sequences were obtained from Bio-Rad. The reactions were emulsified using an auto droplet generator, transferred to 96-well plates, and amplified using the following cycling conditions: 95 ℃ for 10 min; 40 cycles of 94 ℃ for 30 sec, and 55 ℃ for 1 min; 98 ℃ for 10 min. After amplification, plates were read, and fluorescence signals of individual sample droplets were analyzed with a QX200 droplet reader (Bio-Rad). The copy number of the mutant allele frequency relative to that of WT was determined using QuantaSoft V.1.7.4 software (Bio-Rad) by applying a correction based on the Poisson distribution to the number of droplets positive for either mutant or WT DNA. We defined a FAM+ and HEX-, or FAM+ and HEX+ drop as a T790M-positive drop.

**The criteria of *EGFR* gene amplification**

The number of *EGFR* Del19 or L858R mutant copies was detected 2×10^7^, which is the quantitative upper limit of the QX200 Digital Droplet PCR system, in the six samples, and these samples were considered to have *EGFR* gene amplification. The copy number divided by the amount of DNA templates of the 450 samples used in this study is shown in the Supplementary Figure 2. The *EGFR* gene copy number of the six samples predicted to have *EGFR* gene amplification was 1×10^4^ (1.0E+04) copy/ng or more. In contrast, On the other hand, the mean copy number of the *EGFR* gene in the samples excluding these six samples was 5.2×10 (5.2E + 01) copy/ng, and the mean copy of *EGFR* gene in genomic DNA derived from healthy human blood cells used for control was 2.7×10^2^ (2.7E + 02) copy/ng. From these results, we decided the six samples have *EGFR* gene amplification, and excluded from this analysis.

**Primer and probes for mutant F795F or WT F795F**

The sequence was as follows: F795F-FAM; Forward AGGCAGC+C+AAAGGG, WT F795-HEX, A
